# Dietary propolis complementation relieves the physiological and growth deterioration induced by *Flavobacterium columnare* infection in juveniles of common carp (*Cyprinus carpio*)

**DOI:** 10.1371/journal.pone.0292976

**Published:** 2023-10-13

**Authors:** Hesham A. Hassanien, Yousof N. Alrashada, Ahmed O. Abbas, Abdelwahab M. Abdelwahab

**Affiliations:** 1 Department of Animal and Fish Production, College of Agriculture and Food Sciences, King Faisal University, Hofuf, AL-HASA, Saudi Arabia; 2 Animal Production Department, Faculty of Agriculture, Cairo University, Giza, Egypt; 3 Department of Animal Production, Faculty of Agriculture, Fayoum University, Fayoum, Egypt; Kafrelsheikh University, EGYPT

## Abstract

The current study was proposed to explore the role of dietary propolis (PR) supplementation in alleviating the negative effects of columnaris disease (CD) challenge on the growth performance, plasma biochemicals, antioxidant activity, stress indicators, and immunological reactions of common carp (*Cyprinus carpio*) fish. Five hundred forty common carp juveniles were evenly placed in thirty-six 100-L tanks and stocked for acclimatization to the lab conditions with a control diet within a started period of 14 days. Fish (average initial weight of 7.11±0.06 g) were randomly distributed into one of six treatment groups (6 replicate tanks × 15 fish per tank in each treatment group). Fish in the first group was assigned as a negative control without CD challenge or PR supplementation. Fish in the other five groups were challenged with CD by immersion of fish for 60 min into a 10-L water bath supplemented with 6×10^6^ CFU/mL (median lethal dose, LD_50_) of pathogenic *F*. *columnare* bacteria. After infection, the fish were restored to their tanks and fed on a basal diet supplemented with PR at 0, 3, 6, 9, or 12 g/kg diet. The experimental period continued for 6 consecutive weeks in which the feed was introduced twice a day (8:00 and 15:00 h) at a rate of 2% of the fish biomass. Ten percent of water was siphoned and renewed after each meal every day, in addition to 50% of water refreshment after cleaning the tank every three days. The tanks were continuously aerated and provided with standard rearing conditions for carp fish (24.0±1.12°C, 7.7±0.22 pH, 6.3±0.16 mg/L O_2_, and 14L/10D photoperiod). The growth performance traits such as feed intake (FI), weight gain (WG), final weight (FW), specific growth rate (SGR), feed efficiency (FE), and cumulative mortality rates (CM) were recorded during the experimental period. At the end of the trial, blood samples were obtained from the fish to evaluate some plasma biochemicals, including aspartate aminotransaminase (AST), alanine aminotransferase (ALT), creatinine (CRE), alkaline phosphatase (ALP), and lactate dehydrogenase (LDH), antioxidant biomarkers, including total antioxidant capacity (TAOC), total superoxide dismutase (TSOD), reduced glutathione (rGSH), and catalase (CAT), stress indicators, including heterophil to lymphocyte (H/L) ratio, cortisol (COR), malondialdehyde (MDA), and myeloperoxidase (MPO), and immunological reactions, including peripheral blood leukocyte proliferation (PBLP), phagocytosis activity (PHG), lysozyme activity (LYS), alternative complement hemolytic action (ACH50), and total immunoglobulin concentration (TIG). In addition, samples of infected fish gills were taken to quantify the number of *F*. *columnare* in the PR-supplemented groups using the quantitative real-time polymerase chain reaction (qPCR) technique. The results showed that incorporating PR into the dietary ingredients of common carp has a protective effect against the challenge with *F*. *columnare* infection. There were linear and quadratic positive trends (*P* < 0.05) in most parameters of growth performance, plasma biochemicals, antioxidant activity, stress indicators, and immunological reactions with the increased PR-supplemented levels in the diet of infected fish. The best results were obtained when using PR at 9 g/kg in the diet, while higher levels (12 g/kg PR) showed an adverse trend in the evaluated parameters. The FI, WG, FW, SGR, and FE were improved by approximately 37, 104, 34, 73, and 49% in the fish treated with 9 g/kg PR compared to none-PR-infected fish. In addition, adding PR at the 9 g/kg diet level was the best dose that reduced the H/L ratio, COR, MDA, and MPO by about 14, 52, 48, and 29%, respectively, in the infected fish. Furthermore, the mortality rate was reduced by 94%, and the number of pathogenic bacteria cells adherent to the fish gills was lowered by 96% in the infected fish treated with 9 g/kg PR compared to none-PR infected fish. Our results concluded that dietary supplementation with 9 g/kg PR could be a promising nutritional approach for improving the growth performance, physiological profile, and health status of common carp fish, particularly when challenged with *F*. *columnare* or similar bacterial infections.

## Introduction

Aquaculture production of common carp (*Cyprinus carpio*), which is one of the most widely cultured species of freshwater fish, importantly contributes to the world’s protein supply [1]. However, common carp productivity in natural areas and aquaculture farms is frequently challenged by various circumstances, including the spread of diseases. Pathogenic infection with viruses, bacteria, fungi, parasites, etc., may affect fish health, lower growth outputs, and raise management expenses of common carp [2].

Columnaris disease (CD) is caused by *Flavobacterium columnare*, an opportunistic pathogenic bacteria that infects several freshwater fish species [3]. CD typically affects fish on their external surfaces, specifically the gills, fins, and skin, and it may cause systemic infection in severe cases. Lesions ranged in severity from local freckles to small eroded and hyperemic patches that progressed to big inflammation and hemorrhagic necrotic lesions in the underlying muscle tissue [4]. The infectious spread also corresponds to high-stress levels and poor fish health [5]. It was found that *F*. *columnare* infection negatively affected the innate immune response and increased the expression of inflammatory cytokines and stress proteins in carp fish [6, 7]. In addition, some hematological and physiological parameters were inversely influenced by CD in infected fish, e.g., a significant decrease in blood corpuscles, hemoglobin, and lymphocytes, and a significant increase in lactate dehydrogenase, alkaline phosphatase, aspartate aminotransferase, and creatine kinase, in addition to remarkable hyponatremia, hypochloremia and hyperglycemia [8].

Some therapeutic methods, including antibiotic administration and chemical dips, have been widely used to withstand CD outbreaks in some geographic areas. However, the intensive use and residues of such substances in aquatic products brought further attention to their negative attributes on human health [9]. Moreover, it was reported that *F*. *columnare* bacteria isolated from some fish can exert clinical resistance to several important antibiotics such as quinolones and tetracyclines. Therefore, there is an urgent need for innovative approaches to preventing and recovering from CD in the aquaculture of carp and other fish species [4]. Some alternatives have been suggested in this context, such as probiotics, prebiotics, antimicrobial peptides, herbal extracts, and natural resources [10–12].

Honeybees collect a variety of plant resins, waxes, and aromatic oils to create a product called propolis (PR) [13]. This product has been interestingly introduced into fish diets for its effective bioactive materials such as flavonoids and polyphenols compounds [14–16]. Other advantageous qualities of PR include antioxidant capabilities [17], immune stimulation [18], anti-inflammatory activities [19], and antibacterial effects (Kroko et al., 2012). Previous research concluded the beneficial effects of PR administration on cultured fish’s innate immunity, plasma biochemicals, and growth performance [20–24]. In addition, the fish resistance to bacterial infection was evidenced in several investigations [22, 25–28]. Furthermore, PR ameliorated the harmful effects of other thermal and cytotoxic stress factors on fish physiological performance, immune response, and redox status [23, 29, 30].

According to our knowledge, the protection effects of propolis against *F*. *columnare* infection in aquaculture have not been fully recognized and need to be investigated. Therefore, the present research aimed to evaluate the possible ameliorative impact of dietary propolis inclusion on growth and physiological deterioration in common carp suffering from columnaris disease infection.

## Materials and methods

### Ethical statement

This experiment was conducted under the Declaration of Helsinki and approved by the Ethical Research Committee of Saudi Arabia’s King Faisal University (approval code KFU-REC-2023-APRIL-ETHICS117).

### *Flavobacterium columnare* isolation and challenge

Naturally infected carp were collected from a private fish farm and euthanized with an overdose of MS222 (500 mg/L for 10 min) [31]. Bacteriological swabs from skin lesions were inoculated onto *F*. *columnare* growth medium (FCGM) in agar plates and grown for 48 h at 25°C [32]. The pure dominant colonies of *F*. *columnare* were presumptively identified following the Analytical Profile Index API 20E Test [33]. The recovered bacterial cells were re-suspended in sterile phosphate-buffered saline (PBS, pH 7.2), adjusted to a concentration of 10^6^ colony forming unit (CFU) /mL using the pour plate method, and then kept in 30% glycerol at −80°C until used for the challenge study. Fish were challenged by being immersed for 60 min into a water bath (10-L) supplemented with 6 mL (approximately 6×10^6^ CFU/mL) of the retrieved stock of *F*. *columnare* culture after thawing at room temperature [34]. The exposed dose in the present study (6×10^6^ CFU/mL) was calculated to be at a concentration of the median lethal dose (LD_50_) of *F*. *columnare* for the challenge based on preliminary experiments. Briefly, small groups of common carp (15 fish per group) were bathed for 60 min in 10-L buckets containing different concentrations of *F*. *columnare* previously prepared from the retrieved stock culture. The fish mortality rate was recorded for 14 days after infection, and the LD_50_ was estimated as 6×10^6^ CFU/mL.

### Propolis analysis and diet preparation

The PR was gathered as a yellow-brown powder from a beehive station at the King Faisal University of Saudi Arabia’s Agricultural and Veterinary Research Center. The chemical characteristics of three PR samples were examined using the AOAC-adopted techniques [35]. The PR total polyphenolic and flavonoid concentrations were also assayed using the Folin-Ciocalteu and aluminium chloride colorimetric techniques. Gallic acid and quercetin were standard for each assay [36]. According to prior research [37], the 2,2-diphenyl-1-picrylhydrazyl (DPPH) radical scavenging assay was used to identify the PR’s antioxidant activity. Table 1 presents the results obtained from the PR analysis.

**Table 1:** The propolis chemical analysis.

Assay ^1^	Result ^2^
**DM (%)**	90.8 ± 5.07
**Carbohydrate (%)**	1.9 ± 0.07
**Crude fiber (%)**	68.7 ± 3.26
**Total lipids (%)**	9.2 ± 1.31
**Crude protein (%)**	2.6 ± 0.18
**Total ash (%)**	0.9 ± 0.04
**Phenolic content (mg GAE/g)**	162.4 ± 4.75
**Flavonoid content (mg QE/g)**	233.5 ± 9.82
**Antioxidant activity (IC50, μg/mL)**	79.3 ± 2.36

^1^ GAE, gallic acid equivalent; QE, quercetin equivalent; IC50, the sample concentration that achieved 50% inhibition of the 2,2-diphenyl-1-picrylhydrazyl (DPPH) free radicals

^2^ The mean ± standard deviation of three determinations on dry matter (DM) basis.

Based on the conclusion of previous research [20, 22, 24–28], the basal diet ingredients were properly mixed with various levels of PR (0, 3, 6, 9, and 12 g/kg diet) to form the experimental diets. The experimental diets were compacted into 1.5 mm diameter pellets using a manual pelletizer. The diet pellets were then dried at room temperature for 24 hours before, packaged in dark vessels, and kept in a refrigerator at 4°C. Table 2 shows the basal diet’s contents and the nutritional analysis performed by AOAC techniques [35].

**Table 2:** The ingredients and chemical analysis of the basal diet introduced to common carp fish.

Components	g/kg as fed	Nutritional analysis	g/kg (DM basis)
**Soybean meal**	350	**Dry matter (DM)**	894.5
**Yellow corn**	60	**Crude protein (CP)**	340.2
**Fish meal**	150	**Crude lipids (CL)**	102.4
**Wheat bran**	260	**Crude fiber (CF)**	52.3
**Cotton seed meal**	90	**Crude ash (CA)**	58.8
**Vegetable oil**	65	**Nitrogen-free extract (NFE)** ^**2**^	446.3
**Premix** ^**1**^	10	**Gross energy (GE)** ^**3**^	19.7
**Di-calcium phosphate**	5		
**Methionine**	5		
**Lysin**	5		

^1^ Contents per kg of premix: 40000 IU retinol, 4000 IU cholecalciferol, 400 mg α-tocopherol acetate, 12 mg menadione, 30 mg thiamine, 40 mg riboflavin, 30 mg pyridoxine, 80 μg cyanocobalamin, nicotinic acid, 10 mg folic acid, 3 mg biotin, 100 mg pantothenic acid, 500 mg inositol, and 500 mg ascorbic acid, 40 mg manganese sulfate, 10 mg magnesium oxide, 40 mg potassium sulfate, 60 mg zinc carbonate, 0.4 mg potassium iodide, 12 mg Copper sulfate, 250 mg ferric citrate, 0.24 mg sodium selenite, 0.2 mg cobalt

^2^ NFE = 1000 –(CP + CL + CF + CA).

^3^ GE (MJ/kg) = (23.63 CP + 39.52 CL + 17.15 NFE) / 1000.

### Experimental protocols

Five hundred forty common carp juveniles (*Cyprinus carpio*) were provided by a local aquaculture company (Saqua Co., Riyadh, Saudi Arabia). Fish were evenly distributed in thirty-six 100-L tanks (15 fish per tank) and stocked for acclimatization to the lab conditions with a control diet within 14 days. Fish weighed 7.11±0.06 g at the start of the trial. Then, fish in every six tanks were randomly assigned as 6 replicates for one of six treatment groups. Fish in the first group were assigned as a negative control without CD challenge or PR supplementation. Fish in the other five groups were challenged with CD infection (LD_50_ of *F*. *columnare* at 6×10^6^ CFU/mL), as the methods mentioned before. After that, the infected fish were restored to their tanks and fed on a basal diet supplemented with PR at 0, 3, 6, 9, or 12 g/kg, respectively. The experimental period continued for six consecutive weeks. The tanks were continuously aerated using an air blower throughout the experimental period. Twenty percent of the tank’s water was siphoned while collecting feed remains and immediately compensated with fresh water. Besides, the tanks were cleaned every three days from the wastes and renewed with 50% fresh water. The water physiochemical parameters were monitored daily to provide qualified rearing conditions for carp fish, including a temperature of 24.0±1.12°C, pH of 7.7±0.22, dissolved O_2_ of 6.3±0.16 mg/L, and photoperiod of 14 h light and 10 h dark.

### Fish performance

Every other week, the fish bulk weight of each tank was recorded using a digital scale, and the feed amount was adjusted at a rate of 2% of the total biomass. The feed was introduced twice a day at 8:00 and 15:00 h. After one hour of feeding each meal, the leftover feed was collected by siphoning fish tanks. The collected leftover feeds were physically separated from feces using a metal spatula, stored in a freezer at −18°C, dried in a ventilated oven at 55°C, and weighed. After 6 weeks, the total feed intake (FI) was calculated by deducting the total feed remains from the offered feed amounts. The initial (IW) and final (FW) weights of fish were recorded per replicate during the experimental period (6 weeks). Fish weight gain (WG) was then determined (FW–IW). A specific growth rate (SGR) was computed per replicate in each group [(Ln(FW)–Ln(IW))*100/(days)]. The feed efficiency (FE) was determined, given the percentage of WG over the FI for each replicate. The cumulative mortality rate (CM) was also determined by calculating the percentage of dead fish from the total fish number in each tank throughout the experimental period.

### Fish sampling and processing

As soon as the trial ended (after 6 weeks), four fish per replicate in each treatment group were randomly netted and anesthetized by submersion in water containing 0.04% 2-phenoxyethanol [38]. Blood samples were taken from the fish’s caudal vein and immediately dispensed into heparinized sterile microtubes. Two whole blood samples were used for the determination of a stress indicator heterophil to lymphocyte (H/L) ratio and immunological parameters peripheral blood leukocyte proliferation (PBLP) and phagocytosis activity (PHG). The other two blood samples were centrifuged at 2,300 x *g* for 5 min at 4°C to separate the plasma. Plasma samples were then kept in a deep freezer at -20°C until used for one of the other assays, including plasma biochemicals, antioxidant biomarkers, and the remaining parameters of stress indicators and immunological reactions, as detailed below. Furthermore, a section of the left second-gill arch was collected (approximately 50 mg) from one fish per replicate to quantify the *F*. *columnare* bacteria on the fish gill after PR treatment, as mentioned below.

#### Plasma biochemical

The plasma biochemicals, including aspartate aminotransaminase (AST), alanine aminotransferase (ALT), creatinine (CRE), alkaline phosphatase (ALP), and lactate dehydrogenase (LDH), were evaluated according to the manufacturer protocols of colorimetric assay kits (K236, K235, K031, K091, and K046, respectively, Elabscience Biotechnology Inc., Houston, TX, USA). AST and ALT activities allow the formation of oxaloacetic and pyruvic acids, respectively, which in turn react with phenyl hydrazine to form phenylhydrazone, showing a reddish brown color that can be measured at 510 nm [39, 40]. CRE was measured indirectly using the sarcosine oxidase method, which produces a pink compound that can be detected at 515 nm [41]. ALP activity was indirectly calculated following the methods termed by Fernandez and Kidney [42]. ALP decomposes the benzene disodium phosphate into free phenol and phosphoric acid. Phenol reacts with 4-aminopyrine in an alkaline solution and oxidizes by potassium ferricyanide, forming a red quinone derivative that can be measured at 520 nm. LDH catalyzes lactic acid to produce pyruvate in the presence of coenzyme I. The reddish-brown color that elaborates from the pyruvate reaction with phenyl hydrazine could be measured at 540 nm [43]. An automated microplate reader (ELx808TM BioTek Instruments, Winooski, Vermont, USA) was used to scan these assays’ results.

#### Antioxidant biomarkers

The total antioxidant capacity (TAOC), total superoxide dismutase (TSOD), reduced glutathione (rGSH), and catalase (CAT) were evaluated in the collected plasma samples following the protocols of colorimetric kits (K136, K019, K030, and K031, respectively) obtained from Elabscience Biotechnology Inc. (Houston, TX, USA). The TAOC assay was performed according to Uwikor et al. [44]. The method is based on reducing ferric salt reagent by antioxidant enzymes and molecules in the body system, then converting this reaction into a colored product by phenanthroline and measuring it at 520 nm. The TSOD was assayed based on its inhibitory effect on the nitrite formation from hydroxylamine oxidation by the xanthine-xanthine oxidase reaction system. The nitrite could be turned purple by adding a chromogenic reagent and finally measured calorimetrically at 550 nm [45]. The rGSH was determined indirectly through its reaction with dinitrobenzoic acid (DNBT) and releasing a yellow complex which can be measured by colorimetric assay at 405 nm [46]. The CAT activity was also assayed based on the principle that CAT decomposes H2O2. Then, the residual H2O2 reacts with ammonium molybdate, forming a yellowish complex that can be measured at 405 nm [47].

#### Stress indicators

The H/L ratio was measured as a stress indicator according to methods described in a previous study [48]. In brief, a drop of heparinized blood was smeared, air-dried, and processed by Hema-3 stain solutions (Fisher Scientific, Pittsburg, PA, USA). The heterophils and lymphocytes were differentiated and counted in 200 leukocyte cells, then the H/L ratio was calculated. Other stress indicators were also measured in the plasma samples, including cortisol (COR), malondialdehyde (MDA), and myeloperoxidase (MPO). The COR concentration was measured using specific ELISA kits for fish (E08487f, CUSABIO, Wuhan, Hubei, China). Standards or diluted samples (1:100) were added to microtiter plate wells with antibodies specific for Cortisol and Horseradish Peroxidase (HRP) conjugated goat-anti-rabbit antibody to initiate a competitive inhibition reaction between the pre-coated COR and COR in samples. A chromogenic substrate followed by a stop solution was then added to develop a color that can be measured at 450 nm [49]. The MDA was assayed through its reaction with thiobarbituric acid (TBA) and then produced a red compound that can be measured calorimetrically at 532 nm [50]. The MPO was measured by reducing hydrogen peroxide to a complex that reacts with o-dianisidine and produces a yellowish color that can be read at 460 nm [51]. Colorimetric kits obtained from Elabscience Biotechnology Inc. (Houston, TX, USA) were used for the MDA and MPO assays (K025 and K074, respectively).

#### Immunological reactions

The PBLP and PHG assays were performed according to the methods described in a previous study [52]. First, the mononuclear cells were isolated from the heparinized blood samples by centrifuging with a density gradient solution (Histopaque 1077, Sigma-Aldrich, USA). The isolated cells were resuspended with RPMI-1640 complete culture medium (Invitrogen Corp., Grand Island, NY, USA) to a concentration of 3×10^6^ cells/mL of more than 95% viable cells (tested by trypan blue stain). For PBLP assay, lymphocyte proliferation was stimulated by adding lipopolysaccharide solution (10 μg/mL LPS, Sigma, MA, USA), followed by incubation with tetrazolium salt MTT (3-(4,5-dimethyl-2thiazolyl)-2,5-diphenyl tetrazolium bromide), then stimulation index (SI) was determined by calculating the mean ratio of optical density measured at 570 nm for the stimulated and non-stimulated lymphocytes. For PHG assay, the recovered leukocyte suspension was mixed with a stained yeast suspension (*Candida albicans*, Sigma, MA, USA) at a ratio of 1:4, and then by using a light microscope and a hemocytometer, the phagocytosis activity was calculated enumerating the percentage of phagocytes with engulfed yeast of the total phagocytes.

Furthermore, other immunological parameters, such as lysozyme activity (LYS), alternative complement hemolytic action (ACH50), and total immunoglobulin concentration (TIG), were also measured in the plasma. The LYS activity was determined spectrophotometrically at a wavelength of 450 nm using the turbidimetric technique, which is based on the potential of lysozyme proteins existing in the plasma to break down the cell wall of *Micrococcus lysodeikticus* suspension (Sigma-Aldrich, Burlington, MA, USA), according to the methods described in a previous study [22]. The ACH50 and TIG were evaluated according to the protocols cited in a previous study [12]. The plasma volume that produced 50% hemolysis of sheep red blood cells (SRBC) was used to calculate ACH50. The plasma TIG level was measured using the microprotein pyrogallol red assay technique (TP0400 kits, Sigma, MA, USA). The immunoglobulin precipitation was accomplished using a polyethylene glycol solution at a concentration of 12%. Then, the TIG level was provided after deducting the protein content before and after precipitation.

#### *F*. *columnare* quantification in the fish gill

According to the procedures outlined by Beck et al. [34], a quantitative real-time polymerase chain reaction (qRT-PCR) was employed to quantify the *F*. *columnare* adherent to the gill of the fish in all the treatment groups. DNA extraction was carried out using a DNeasy Blood and Tissue Kit from Qiagen per the manufacturer’s guidelines. The primers and FAM probe (Table 3), which were applied to target a portion of the *F*. *columnare* chondroitin AC lyase gene, were designed according to Panangala et al. [53]. A positive control consisted of a standard extracted template (1 × 10^4^ CFU/mL), and a negative control without extracted template was included in each run of the Q-PCR reactions. All samples were run in duplicate, and the reaction components and protocols of the qPCR were performed on a SimpliAmp Thermal Cycler (Thermo Fisher Scientific Inc., MA, USA) by using SuperScript^TM^ III One-Step PCR System with Platinum^®^ Taq DNA Polymerase kit (Invitrogen, Thermo Fisher Scientific, MA, USA). For normalization, the qPCR data were divided by the amount of template DNA put into each reaction, and the results are reported as CFU/ng of template DNA. Before counting the CFUs in a sample, a standard curve was created by plotting the qPCR data of bacteria serial dilutions against the CFU previously counted for each dilution.

**Table 3:** Primers and FAM probe sequences for qRT-PCR analysis of *F*. *columnare* bacteria^1^.

Item	Sequence (5’-3’)	Size (bp)	Melting °C
Forward primer	CCTGTACCTAATTGGGGAAAAGAGG	25	57.4
Reverse primer	GCGGTTATGGCCTTGTTTATCATAGA	25	55.7
Probe	ACAACAATGATTTTGCAGGAGGAGTATCTGATGGG	35	68.8

^1^ The primers and probe target a region of the chondroitin AC lyase gene of *F*. *columnare* (GenBank accession number AY912281).

### Statistical analysis

The statistical analysis was performed using IBM SPSS Statistics version 22 (IBM Corp., NY, USA). All the data were checked for normal distribution before the statistical analysis. An independent t-test was used to compare infected fish’s data vs. those non-infected without propolis supplementation. The remaining data for infected fish that received various levels of propolis (0, 3, 6, 9, and 12 g/kg diet) were analyzed using one-way analysis of variance (ANOVA). The results obtained were expressed as mean ± standard error (SE), and Duncan’s multiple comparison test explored the significant differences between treatment means. Each replicate tank was assigned as an experimental unit for the growth parameters (*n* = 6). The fish gills were considered experimental units for the qRT-PCR assay (*n* = 6). Meanwhile, fish bold/plasma samples were the experimental units for the other measurements (*n* = 12). Significant values were marked at p < 0.05.

## Results

### Fish performance

The effects of the different dietary supplementation levels of PR on the growth performance of common carp fish infected with CD are presented in Table 4. Growth performance was negatively influenced by *F*. *columnare* infection in the control groups (without PR supplementation). FI was significantly (*P* < 0.05) reduced by 18% in the challenged group compared to the none-challenged group with no effect of PR supplementation. The fish WG and FW were significantly (*P* < 0.05) reduced by 44% and 22%, respectively. Also, the SGR and FE were significantly (*P* < 0.05) reduced by 34% and 31%, respectively. When PR was supplemented to the infected fish, significant (*P* < 0.05) linear and quadratic effects were observed on the growth performance traits with increasing PR levels in the diets. Dietary PR supplementation in the infected fish up to 9 g/kg significantly (*P* < 0.05) induced the maximum improvement in the FI, WG, FW, SGR, and FE by approximately 37, 104, 34, 73, and 49%, respectively, compared to the infected fish without PR supplementation. Higher PR levels (12 g/kg) did not significantly affect the growth performance compared to non-treated PR in the infected fish. On the other hand, the CM of challenged fish without propolis supplementation increased to 52% compared to non-challenged fish (*P* < 0.05). However, PR supplementation at increasing levels in the diets linearly and quadratically(*P* < 0.05) reduced the CM of infected fish, recording less than 5% in the group treated with 9 g/kg PR (Table 4).

**Table 4:** Effect of dietary propolis (PR) supplementation levels on the productive performance of common carp fish infected with columnaris disease (CD).

CD challenge	PR levels (g/kg diet)	IW, g	FW, g	WG, g	SGR, %	FI, g	FE, %	CM, %
**Not infected**	**0**	7.4 ± 0.18	13.4 ± 0.14	6.0 ± 0.15	1.4 ± 0.05	11.2 ± 0.18	53.5 ± 1.78	0.0 ± 0.00
**Infected**	**0**	7.1 ± 0.22	10.4 ± 0.28 ^d^	3.4 ± 0.10 ^d^	0.9 ± 0.02 ^d^	9.2 ± 0.17 ^c^	36.7 ± 0.87 ^c^	52.2 ± 2.05 ^a^
	**3**	7.3 ± 0.16	11.7 ± 0.10 ^c^	4.4 ± 0.18 ^c^	1.1 ± 0.05 ^c^	11.0 ± 0.58 ^b^	40.4 ± 1.57 ^bc^	38.9 ± 2.05 ^b^
	**6**	7.2 ± 0.17	12.7 ± 0.25 ^b^	5.5 ± 0.35 ^b^	1.3 ± 0.09 ^b^	11.8 ± 0.32 ^ab^	46.8 ± 3.75 ^b^	12.2 ± 4.01 ^c^
	**9**	7.1 ± 0.14	14.0 ± 0.21 ^a^	6.9 ± 0.21 ^a^	1.6 ± 0.05 ^a^	12.6 ± 0.07 ^a^	54.7 ± 1.73 ^a^	4.4 ± 2.22 ^c^
	**12**	6.9 ± 0.17	10.4 ± 0.28 ^d^	3.6 ± 0.33 ^d^	1.0 ± 0.09 ^cd^	9.5 ± 0.24 ^c^	37.6 ± 3.67 ^c^	5.6 ± 2.05 ^c^
***P*-value**								
**Infected *vs*. not infected**		0.310	<0.001	<0.001	<0.001	<0.001	<0.001	<0.001
**PR effect in infected fish**		0.583	<0.001	<0.001	<0.001	<0.001	<0.001	<0.001
**PR-Linear term**		0.346	0.004	0.001	0.006	0.035	0.059	<0.001
**PR-Quadratic term**		0.188	<0.001	<0.001	<0.001	<0.001	<0.001	<0.001

Data are presented as mean ± SE for ten fish in each tank as a replicate (*n* = 6 tanks per treatment group). Means within the same column with different superscripts significantly differ (*p* < 0.05). IW, initial weight; FW, final weight; WG, weight gain; SGR, specific growth rate; FI, feed intake; FE, feed efficiency; CM, cumulative mortality.

### Blood biochemicals

Fish plasma biochemicals of the different experimental groups are shown in Table 5. The results demonstrated that CD challenge without PR treatment significantly (*P* < 0.05) elevated the AST, ALT, LDH activities, and CRE levels compared to the non-challenged fish group. Propolis supplementation generally ameliorated the negative impact of *F*. *columnare* infection on the plasma biochemical parameters. Increasing the levels of PR in the diet of infected fish was able to linearly and quadratically (*P* < 0.05) decrease the AST and ALT activities and linearly (*P* < 0.05) decrease the plasma CRE, ALP, and LDH.

**Table 5:** Effect of dietary propolis (PR) supplementation levels on the blood biochemicals of common carp fish infected with columnaris disease (CD).

CD challenge	PR levels (g/kg diet)	AST, IU/L	ALT, IU/L	CRE, μmol/L	ALP, U/L	LDH, U/L
**Not infected**	**0**	46.7 ± 1.74	15.1 ± 0.49	30.6 ± 1.90	44.3 ± 2.34	143.3 ± 2.29
**Infected**	**0**	84.7 ± 4.15 ^a^	32.2 ± 1.37 ^a^	68.1 ± 4.60 ^a^	47.2 ± 2.27 ^a^	162.2 ± 2.72 ^a^
	**3**	71.7 ± 5.67 ^ab^	26.1 ± 1.52 ^b^	67.4 ± 6.71 ^a^	43.7 ± 1.99 ^a^	161.4 ± 3.21 ^a^
	**6**	64.3 ± 6.53 ^b^	19.1 ± 1.38 ^c^	50.76 ± 5.59 ^b^	37.8 ± 1.51 ^b^	154.1 ± 3.38 ^ab^
	**9**	47.0 ± 3.81 ^c^	16.5 ± 1.20 ^c^	42.1 ± 4.88 ^bc^	37.7 ± 1.90 ^b^	146.5 ± 4.85 ^b^
	**12**	59.9 ± 3.54 ^bc^	18.1 ± 2.03 ^c^	33.8 ± 4.06 ^c^	37.2 ± 1.20 ^b^	148.3 ± 4.76 ^b^
**P-value**						
**Infected vs. not infected**		<0.001	<0.001	<0.001	0.388	<0.001
**PR effect in infected fish**		<0.001	<0.001	<0.001	<0.001	0.014
**PR-Linear term**		<0.001	<0.001	<0.001	<0.001	0.001
**PR-Quadratic term**		0.026	<0.001	0.708	0.086	0.746

Data are presented as mean ± SE for 6 replicates with 2 fish per replicate (*n* = 12 samples per treatment group). Means within the same column with different superscripts significantly differ (*p* < 0.05). AST, aspartate aminotransaminase; ALT, alanine aminotransferase; CRE, creatinine; ALP, alkaline phosphatase; LDH, lactate dehydrogenase.

### Antioxidant biomarkers

The antioxidant biomarkers of carp fish infected with *F*. *columnare* and treated with different levels of PR are presented in Table 6. The CD challenge disturbed the antioxidant system of the fish, recording a significant (*P* < 0.05) reduction by 17, 47, 26, 27, and 41% in the TAOC, TSOD, rGSH, and CAT biomarkers, respectively, when compared with non-challenged fish. In contrast, PR supplementation, at all experimented levels, modulated the negative impact of *F*. *columnare* on fish antioxidant biomarkers. A linear effect (*P* < 0.05) for increasing the dietary PR level was evidenced in the antioxidant biomarkers of infected fish, and the best improvement in the antioxidant biomarkers was obtained with 6–9 g/kg PR compared to the other levels.

**Table 6:** Effect of dietary propolis (PR) supplementation levels on the antioxidant enzyme activity of common carp fish infected with columnaris disease (CD).

CD challenge	PR levels (g/kg diet)	TAOC, U/mL	TSOD, U/mL	rGSH, μmol/L	CAT, U/mL
**Not infected**	**0**	1.4 ± 0.10	7.2 ± 0.23	16.6 ± 0.22	52.5 ± 0.88
**Infected**	**0**	1.2 ± 0.02 ^b^	3.8 ± 0.23 ^c^	12.2 ± 0.22 ^d^	38.4 ± 1.03 ^b^
	**3**	1.2 ± 0.04 ^b^	6.3 ± 0.30 ^b^	14.3 ± 0.12 ^c^	39.6 ± 0.87 ^ab^
	**6**	1.3 ± 0.02 ^a^	7.3 ± 0.17 ^a^	15.5 ± 0.06 ^b^	41.2 ± 0.64 ^a^
	**9**	1.4 ± 0.025 ^a^	7.0 ± 0.39 ^ab^	16.0 ± 0.10 ^a^	42.3 ± 1.13 ^a^
	**12**	1.2 ± 0.03 ^b^	6.2 ± 0.40 ^b^	14.3 ± 0.21 ^c^	42.0 ± 0.73 ^a^
***P*-value**					
**Infected *vs*. not infected**		0.027	<0.001	<0.001	<0.001
**PR effect in infected fish**		<0.001	<0.001	<0.001	0.017
**PR-Linear term**		0.019	<0.001	<0.001	0.001
**PR-Quadratic term**		<0.001	<0.001	<0.001	0.291

Data are presented as mean ± SE for 6 replicates with 2 fish per replicate (*n* = 12 samples per treatment group). Means within the same column with different superscripts significantly differ (*p* < 0.05). TAOC, total antioxidant capacity; TSOD, total superoxide dismutase; rGSH, reduced glutathione; CAT, catalase.

### Stress indicators

The results of stress indicators evaluated in the common carp fish infected with *F*. *columnare* and supplemented with PR are shown in Table 7. Compared to the control group (no challenge without propolis), *F*. *columnare* infection elevated (*P* < 0.05) the stress indicators by 1.5-fold in the H/L ratio and approximately 2-fold in the COR, MDA, and MPO levels. In the infected fish, PR supplementation significantly (*P* < 0.05) revoked the stress indicators experienced by the fish, displaying a linear and quadratic reduction effect by increasing the levels of PR in the fish diets. Results demonstrated that adding PR at the 9 g/kg diet was the best dose that reduced the H/L ratio, COR, MDA, and MPO levels by about 14%, 52%, 48%, and 29%, respectively.

**Table 7:** Effect of dietary propolis (PR) supplementation levels on the stress indicators of common carp fish infected with columnaris disease (CD).

CD challenge	PR levels (g/kg diet)	H/L ratio	COR, ng/mL	MDA, mg/dL	MPO, mg/dL
**Not infected**	**0**	0.41 ± 0.005	26.5 ± 1.07	2.8 ± 0.09	42.0 ± 0.73
**Infected**	**0**	0.61 ± 0.002 ^a^	54.3 ± 2.16 ^a^	5.9 ± 0.27 ^a^	86.6 ± 0.67 ^a^
	**3**	0.59 ± 0.002 ^b^	46.0 ± 2.51 ^b^	5.2 ± 0.41 ^a^	82.7 ± 0.39 ^b^
	**6**	0.55 ± 0.004 ^c^	36.9 ± 2.51 ^c^	3.9 ± 0.37 ^bc^	74.6 ± 0.79 ^c^
	**9**	0.53 ± 0.008 ^c^	26.2 ± 2.49 ^d^	3.0 ± 0.23 ^c^	61.8 ± 1.03 ^d^
	**12**	0.54 ± 0.012 ^c^	30.6 ± 3.29 ^cd^	4.2 ± 0.22 ^b^	63.5 ± 1.03 ^d^
***P*-value**					
**Infected *vs*. not infected**		<0.001	<0.001	<0.001	<0.001
**PR effect in infected fish**		<0.001	<0.001	<0.001	<0.001
**PR-Linear term**		<0.001	<0.001	<0.001	<0.001
**PR-Quadratic term**		0.019	<0.001	0.034	0.001

Data are presented as mean ± SE for 6 replicates with 2 fish per replicate (*n* = 12 samples per treatment group). Means within the same column with different superscripts significantly differ (*p* < 0.05). H/L ratio, heterophil to lymphocyte ratio; COR, cortisol; MPO, myeloperoxidase; MDA, malondialdehyde.

### Immunological reactions

Table 8 shows the results of immunological reactions in the different experimental groups. *F*. *columnare* infection significantly (*P* < 0.05) impaired all immunological reactions assayed compared to non-infected fish without PR. The reduction amounted to about 60, 43, 22, 58, and 56% in the PBLP, PHG, LYS, ACH50, and TIG, respectively, after fish infection. Upon PR supplementation in infected fish, the reduction in the immunological reactions was significantly (*P* < 0.05) ameliorated. The immunological reactions of infected fish were linearly (*P* < 0.05) enhanced with increasing the levels of PR in the fish diets up to 9 g/kg. In comparison, they started lowering again when using higher levels of PR (12 g/kg).

**Table 8:** Effect of dietary propolis (PR) supplementation levels on the immune status of common carp fish infected with columnaris disease (CD).

CD challenge	PR levels (g/kg diet)	PBLP, SI	PHG, %	LYS, U/mL	ACH50, U/mL	TIG, mg/mL
**Not infected**	**0**	3.0 ± 0.02	16.3 ± 0.18	31.0 ± 0.65	52.0 ± 0.37	3.9 ± 0.01
**Infected**	**0**	1.2 ± 0.01 ^e^	9.3 ± 0.10 ^e^	24.3 ± 0.31 ^c^	21.9 ± 0.08 ^d^	1.7 ± 0.01 ^d^
	**3**	1.3 ± 0.02 ^d^	9.7 ± 0.05 ^d^	24.9 ± 0.75 ^c^	21.4 ± 0.10 ^d^	1.7 ± 0.01 ^d^
	**6**	1.5 ± 0.01 ^c^	10.3 ± 0.03 ^b^	27.8 ± 0.08 ^b^	32.1 ± 0.33 ^b^	2.3 ± 0.02 ^c^
	**9**	1.8 ± 0.01 ^a^	13.6 ± 0.07 ^a^	30.4 ± 0.14 ^a^	36.1 ± 0.13 ^a^	3.2 ± 0.03 ^a^
	**12**	1.6 ± 0.02 ^b^	9.9 ± 0.05 ^c^	28.8 ± 0.29 ^b^	23.3 ± 0.22 ^c^	2.6 ± 0.03 ^b^
**P-value**						
**Infected vs. not infected**		<0.001	<0.001	<0.001	<0.001	<0.001
**PR effect in infected fish**		<0.001	<0.001	<0.001	<0.001	<0.001
**PR-Linear term**		<0.001	<0.001	<0.001	<0.001	<0.001
**PR-Quadratic term**		<0.001	<0.001	0.002	<0.001	<0.001

Data are presented as mean ± SE for 6 replicates with 2 fish per replicate (*n* = 12 samples per treatment group). Means within the same column with different superscripts significantly differ (*p* < 0.05). PBLP, peripheral blood leukocyte proliferation (SI, stimulation index); PHG, phagocytosis activity; LYS, lysozyme activity; ACH50, alternative complement hemolytic action; TIG, total immunoglobulins.

### Quantitative-PCR assay for *F*. *columnare* in fish gill

**Fig 1:**
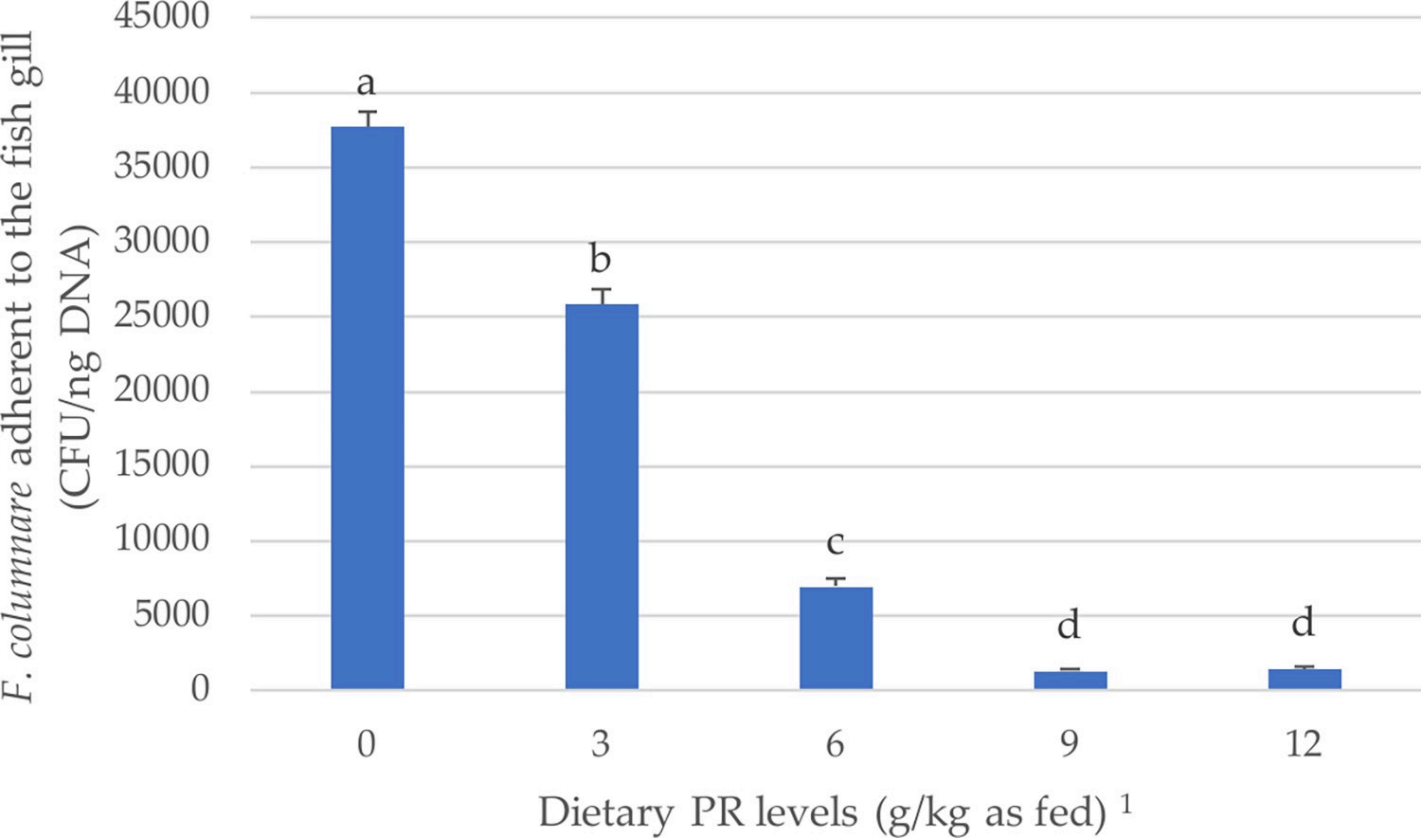
Quantitative polymerase chain reaction (qPCR) assay for *F*. *columnare* attached on the gill of infected fish after the treatment with different levels of propolis (PR). Bars express the means ± SE (*n* = 6 replicate gills per treatment group). Bars with different letters denote significant differences between PR-group means (*P* < 0.05). ^1^ A significant linear and quadratic effect was evidenced for increasing the dietary PR levels in the infected fish (*P* < 0.05).

The effect of PR supplementation levels on the *F*. *columnare* adherent to the gill of infected fish is illustrated in Fig 1. The results from the qPCR analysis confirmed the absence of *F*. *columnare* bacteria in the control group (no challenge and no PR treatment). In contrast, the challenged group untreated with PR had a high concentration of *F*. *columnare* bacteria (37.72±1.011 × 10^3^ CFU/ng DNA) attached to the gill. Dietary PR supplementation at increasing levels (3–12 g/kg) linearly and quadratically (*P* < 0.05) lowered the number of *F*. *columnare* cells attached to the gill of infected fish. The lowest number of bacteria cells was found in the gill of infected fish treated with 9 and 12 g/kg PR (1.28±0.107 and 1.43±0.128 × 10^3^ CFU/ng DNA, respectively) compared to the other PR groups (*P* < 0.05).

## Discussion

In aquatic animals, few studies investigated the positive effects of dietary supplementation with different levels of propolis and its extracts, ranging from 1 to 40 g/kg, on fish performance and resistance to pathogenic bacteria [20, 22, 24–28]. Based on these studies, it was concluded that increasing propolis levels in fish diets up to 10 g/kg linearly improved fish growth, immunity, and disease resistance, while higher doses of PR may induce negative impacts on fish performance [22]. Therefore, the doses of propolis selected to be added to the fish diet in the present study were 0, 3, 6, 9, and 12 g/kg. To the best of our knowledge, the current study provides the first results regarding the efficacy of dietary PR supplementation to mitigate the harmful effects of CD infection in common carp.

The results of the present study demonstrated the severe harmful effects of *F*. *columnare* infection on fish performance. In addition to the external lesions seen on fish fins and skin in the infected groups, the qPCR assay confirmed the presence of high colonies of bacteria cells on the fish gill (37.72×10^3^ CFU/ng DNA), compared to the control group (Fig 1). This PCR technique is specific and sensitive for detecting and quantitating very low levels of *F*. *columnare* in fish gill tissues [54]. The growth parameters and feed efficiency deteriorated, and the mortality rate was 52% in the CD-challenged group (Table 4). Other studies recorded various mortality rates that reached 22% [34], 34% [55], and 80% [56] after challenge, and this variation in mortality could be related to infection severity, lesions, host, and experimental conditions [4]. Moreover, all physiological parameters measured in the current study were disturbed, leading to poor immunity, low antioxidant activity, and high-stress experience in the infected fish. Increasing the AST, ALT, ALP, and LDH levels in the infected fish could be a signal to hepatocyte or muscle cell damage [57, 58], while the high levels of CRE could be a marker of renal dysfunction [59]. The increased ALP and LDH in the infected fish could be attributed to the body response to stress [60]. Like our results, Harikrishnan et al. [55] found that challenge with *F*. *columnare* infection in *Labeo rohita* fish impaired the hemato-biochemical indices, antioxidant system, innate-adaptive immunity, and immuno-antioxidant gene expression; however, this impairment was not significant in comparison with the unchallenged fish in this study. It was reported that *F*. *columnare* pathogenic cells excrete degrading enzymes (particularly AC lyase) and other toxins after attaching to the host tissue, and consequently, overcome the immune defense and disturb the cellular functions of the host [8].

Our results show that dietary supplementation of PR at a dose of 9 g/kg in the infected fish improved the growth performance as indicated by enhancement of 104, 34, 73, and 49% in the WG, FW, SGR, and FE, respectively (Table 4). In *Anguilla japonica*, a similar effect was reported when PR was added to fish diets at lower levels (2.5–5 g/kg), resulting in better growth performance, including WG, SGR, and FE [22]. The positive effect of PR on growth performance may be due to the nutritional value of PR as an additional source of protein, lipids, and carbohydrates in fish diets (Table 1). In addition, the plasma biochemical parameters of AST, ALT, CRE, ALP, and LDH were also decreased with increasing the PR level in the fish diet, suggesting the potential capacity of PR to protect liver, kidney, and muscle tissues of the fish from damage induced by CD infection [61]. It has been evidenced that PR protects against tissue injury of the liver, kidney, and lung in rats [62, 63]. PR analysis shows that it contains a major amount of phenolic and flavonoid compounds with IC50 measurement of about 79 μg/mL, which exerted a strong antioxidant activity [64]. Such PR properties facilitated the reduction in the plasma TAOC, rGSH, TSOD, and CAT activities induced by CD infection (Table 6). The antioxidant properties of propolis can contribute to the MDA decrement and the GSH, CAT, and SOD increments in the infected fish in the present study and other studies [65, 66]. Similarly, the polyphenols and flavonoids extracted from other natural resources, such as *Alchemilla vulgaris* or ulvan seaweed, reduce the oxidative damage and enhance the antioxidant capacity in fish infected with *F*. *columnare* bacteria [55, 56]. These polyphenolic compounds activate various biological pathways, such as eliminating free radicals, decreasing lipoperoxidation, increasing the endogenous enzymatic defenses, and modulation of cell signaling and gene expression; therefore, they enhance the antioxidant and detoxification effects and reduce the symptoms of some diseases [67].

Our results also indicated that some stress indicators, which were elevated by *F*. *columnare* infection, were linearly lowered by increasing the PR supplementation levels (Table 7). [65, 66]Cortisol is the major stress hormone in fish [68]. Consequently, CORT increases the heterophils while decreasing the lymphocytes in blood samples, inducing a high H/L ratio during stress [69]. Thus, PR remarkably decreased the CORT and H/L ratio level in the infected fish in the current study. Although MPO is known as a generator of antimicrobial and immunomodulatory compounds in some leukocyte cells, it could also be considered as a local mediator of tissue damage and measurement of inflammation during disease stress [70]. In the present study, feeding on PR-supplied diets caused a reduction in the plasma MPO activity. This result may be correlated to the anti-inflammatory compounds of PR, such as phenolics, saponins, flavonoids, alkaloids, glycosides, steroids, and terpenoids [19]. It was also reported that targeting MPO with inhibitors or natural compounds attenuated the oxidative stress and inflammation induced by some diseases [71].

Importantly, PR has been used as a complementary or alternative agent to induce immunomodulation in human and animal species [18, 72–75]. In fish species, a substantial improvement in innate immune responses has been obtained after feeding with crude propolis [22, 76]. Our study demonstrated that PR supplementation to common carp diets ameliorated the immune disorders after exposure to CD infection (Table 8). The infected carp supplemented with 6–9 g/kg PR in the current study noticeably improved the PBLP, PHG, LYS, ACH50, and TIG by approximately 24–54%, 10–46%, 14–25%, 46–65%, and 35–86%, respectively, compared to the infected carp untreated with PR. Similarly, PR improved the resistance of channel catfish against *Streptococcus iniae* through stimulation of cellular and humoral immune responses, increasing antibody production, and promoting lysozyme activity [77]. Several experiments applied other successful methods for propolis administration in fish rather than diets to enhance immune response, e.g., intraperitoneal administration at 5 mg/mL in gilthead sea bream [78], 15 mg/mL in turbot [79], or 50 mg/mL in carp [80]. The positive effect of natural products like PR on leukocyte proliferation and immunoglobulin generation in infected fish has a crucial role in disease resistance [81, 82]. Studies have also shown that increasing blood lysozymes and hemolytic complement proteins can support fish health and resistance against bacterial challenge [83, 84].

In the present study, PR was supplemented to the diets of infected fish at increasing levels (3–12 g/kg). The qPCR analysis for *F*. *columnare* cells attached to the gills of infected fish demonstrated that increasing PR levels in the diet linearly decreased the number of bacteria cells in the gills, obtaining approximately 96% lower bacterial cells in the 9 and 12 g/kg PR groups than in the infected group without PR. A linear improvement in the growth, immunity, antioxidant capacity, and stress indicators was obtained by increasing the PR levels in the diets, recording the best results at 9 g/kg. These parameters were decreased again when increasing the PR level to 12 g/kg in the fish diets. Therefore, it could be suggested that the ideal amount of PR supplementation in common carp diets to achieve the most protection against *F*. *columnare* infection is 9 g/kg and that caution should be taken to avoid the negative effects of excess supplementing on fish growth and health.

## Conclusions

The inclusion of PR into the dietary ingredients of common carp showed a protective effect against the challenge of *F*. *columnare* infection. There were linear positive trends in most parameters of growth performance, plasma biochemicals, antioxidant activity, stress indicators, and immunological reactions with the increased PR levels in the diet of infected fish. The best results were obtained using PR at 9 g/kg in the diet. The number of pathogenic bacteria cells adherent to the fish gills was reduced by 96% in infected fish treated with 9 g/kg PR compared to none-PR infected fish. Our results concluded that dietary supplementation with 9 g/kg PR could be a promising nutritional approach to boost the growth performance, physiological profile, and health status of common carp fish, particularly when challenged with *F*. *columnare* or other bacterial infections. Some bioactive compounds in the PR, such as polyphenols and flavonoids, may be responsible for improving aquaculture’s performance and disease resistance. However, the mode of action of PR components needs to be more explored using specific studies comprising transcriptomic, proteomic, and metabolic applications.

## Supporting information

S1 Data (PDF)
