# Supplementary material for: Dietary propolis complementation relieves the physiological and growth deterioration induced by Flavobacterium columnare infection in juveniles of common carp (Cyprinus carpio)
Source: PLoS One. 2023 Oct 13;18(10):e0292976. doi: 10.1371/journal.pone.0292976 (PMC10575500; doi:10.1371/journal.pone.0292976)
Supplement: S1 Data — (PDF) [file pone.0292976.s001.pdf]

NO	GROUP	Challenge	Propolis	IW	FW	WG	WGR	SGR
1	CNT	0	0	7.68	13.25	5.58	72.64	1.30007
2	CNT	0	0	7.75	13.87	6.12	78.99	1.386044
3	CNT	0	0	6.88	13.45	6.58	95.64	1.597827
4	CNT	0	0	6.75	12.80	6.05	89.63	1.523578
5	CNT	0	0	7.75	13.37	5.62	72.53	1.298637
6	CNT	0	0	7.45	13.45	6.00	80.54	1.406584
1	CH0	1	0	7.15	10.48	3.33	46.60	0.910859
2	CH0	1	0	6.88	9.93	3.05	44.42	0.875059
3	CH0	1	0	7.75	11.48	3.73	48.16	0.935953
4	CH0	1	0	6.45	9.68	3.23	50.00	0.965393
5	CH0	1	0	7.63	11.04	3.41	44.73	0.880249
6	CH0	1	0	6.55	10.04	3.49	53.22	1.015917
1	CH3	1	3	7.33	11.48	4.16	56.75	1.070238
2	CH3	1	3	7.20	11.48	4.28	59.47	1.111219
3	CH3	1	3	7.75	12.04	4.29	55.30	1.048061
4	CH3	1	3	7.63	11.59	3.96	51.99	0.996783
5	CH3	1	3	6.77	11.93	5.16	76.20	1.348656
6	CH3	1	3	6.88	11.59	4.71	68.57	1.243308
1	CH6	1	6	7.75	12.82	5.07	65.44	1.198631
2	CH6	1	6	6.88	11.93	5.05	73.51	1.312012
3	CH6	1	6	7.48	12.38	4.90	65.55	1.200272
4	CH6	1	6	7.26	12.38	5.12	70.57	1.271399
5	CH6	1	6	7.18	12.82	5.65	78.70	1.382179
6	CH6	1	6	6.55	13.71	7.16	109.38	1.75946
1	CH9	1	9	6.75	13.71	6.96	103.17	1.687847
2	CH9	1	9	6.88	13.89	7.02	102.08	1.67496
3	CH9	1	9	7.75	14.07	6.32	81.57	1.420128
4	CH9	1	9	7.08	13.36	6.28	88.79	1.513057
5	CH9	1	9	7.28	14.82	7.55	103.73	1.694358
6	CH9	1	9	7.05	14.36	7.31	103.65	1.693381
1	CH12	1	12	6.55	9.68	3.13	47.71	0.928762
2	CH12	1	12	6.78	11.04	4.26	62.89	1.16166
3	CH12	1	12	7.45	10.04	2.59	34.71	0.709372
4	CH12	1	12	7.08	11.48	4.41	62.29	1.152918
5	CH12	1	12	6.28	10.48	4.21	67.05	1.221666
6	CH12	1	12	7.05	9.93	2.88	40.83	0.815212

FI	FCR	FE	Death	Mortality	CF	CFU	Death	Mortality
10.97525	1.97	50.80	1	10	0.001	0	0	0.00
11.84643	1.94	51.67	0	0	0.001	0	0	0.00
10.63125	1.62	61.85	0	0	0.002	0	0	0.00
11.625	1.92	52.04	0	0	0.001	0	0	0.00
11.22643	2.00	50.07	0	0	0.001	0	0	0.00
11.04	1.84	54.35	0	0	0.002	0	0	0.00
8.543527	2.56	39.00	6	60	0.001	37700	8	53.33
9.196429	3.01	33.20	5	50	0.001	39400	7	46.67
9.882813	2.65	37.76	5	50	0.002	35600	7	46.67
9.196429	2.85	35.07	6	60	0.001	41100	8	53.33
9.196429	2.70	37.09	7	70	0.002	38200	9	60.00
9.196429	2.64	37.90	6	60	0.001	34300	8	53.33
9.882813	2.38	42.06	4	40	0.001	23000	6	40.00
9.196429	2.15	46.56	5	50	0.001	29300	7	46.67
10.60268	2.47	40.42	3	30	0.001	25000	5	33.33
11.35603	2.86	34.91	4	40	0.001	23500	6	40.00
12.96317	2.51	39.79	3	30	0.001	27400	5	33.33
12.14286	2.58	38.82	4	40	0.001	26800	6	40.00
12.14286	2.39	41.76	1	10	0.001	6000	2	13.33
12.46696	2.47	40.54	0	0	0.001	5200	1	6.67
12.79643	2.61	38.29	0	0	0.001	8300	0	0.00
11.51071	2.25	44.48	1	10	0.001	8600	2	13.33
10.60268	1.88	53.25	2	20	0.001	6400	3	20.00
11.51071	1.61	62.24	0	0	0.001	7100	1	6.67
12.79643	1.84	54.42	0	0	0.001	900	0	0.00
12.46696	1.78	56.29	0	0	0.001	1600	1	6.67
12.46696	1.97	50.71	1	10	0.001	1100	2	13.33
12.79643	2.04	49.09	1	10	0.001	1500	1	6.67
12.46696	1.65	60.53	0	0	0.001	1400	0	0.00
12.79643	1.75	57.10	0	0	0.001	1200	0	0.00
9.196429	2.94	33.98	2	20	0.001	1200	2	13.33
9.196429	2.16	46.33	1	10	0.002	1300	1	6.67
9.196429	3.56	28.12	0	0	0.001	1000	0	0.00
9.882813	2.24	44.59	0	0	0.001	1700	0	0.00
9.196429	2.19	45.75	1	10	0.001	1800	1	6.67
10.60268	3.68	27.15	1	10	0.001	1600	1	6.67

NO	GROUP	Challenge	Propolis	AST	ALT	CRE	ALP	LDH
1	CNT	0	0	51.94	15.09	32.75	55.19	149.50
2	CNT	0	0	47.35	17.97	42.42	54.17	150.92
3	CNT	0	0	50.53	14.51	32.51	57.89	138.08
4	CNT	0	0	45.14	11.91	23.44	34.45	126.28
5	CNT	0	0	37.08	15.39	24.73	37.28	143.27
6	CNT	0	0	51.42	16.25	36.32	42.59	147.57
7	CNT	0	0	35.06	14.14	22.19	40.14	135.47
8	CNT	0	0	47.33	13.96	26.85	44.17	135.09
9	CNT	0	0	44.53	15.60	29.74	33.71	145.16
10	CNT	0	0	44.59	15.18	28.51	39.18	149.29
11	CNT	0	0	55.86	13.60	27.35	48.64	146.89
12	CNT	0	0	50.11	17.75	41.02	44.39	152.16
1	CH0	1	0	96.02	33.30	78.54	43.32	166.21
2	CH0	1	0	65.91	37.55	61.55	46.86	168.91
3	CH0	1	0	93.41	27.60	62.24	39.26	158.03
4	CH0	1	0	103.56	33.87	87.93	53.29	165.27
5	CH0	1	0	82.17	27.33	54.19	61.26	157.40
6	CH0	1	0	63.56	29.48	48.21	41.13	152.85
7	CH0	1	0	98.81	34.48	80.40	38.04	171.22
8	CH0	1	0	80.14	37.95	75.61	51.59	170.70
9	CH0	1	0	95.08	35.41	82.55	47.68	169.54
10	CH0	1	0	89.45	32.61	74.35	46.79	160.96
11	CH0	1	0	61.08	21.85	34.82	59.10	138.68
12	CH0	1	0	86.83	35.10	76.33	37.93	166.15
1	CH3	1	3	83.43	28.33	85.31	49.35	166.86
2	CH3	1	3	63.27	31.08	72.62	31.75	169.52
3	CH3	1	3	94.48	22.75	78.76	50.28	156.19
4	CH3	1	3	71.22	20.58	53.71	50.05	150.02
5	CH3	1	3	76.02	26.77	74.02	49.06	162.76
6	CH3	1	3	51.15	28.86	52.19	35.12	166.15
7	CH3	1	3	48.64	25.37	44.06	52.08	158.68
8	CH3	1	3	68.86	16.09	42.05	36.73	139.30
9	CH3	1	3	42.08	26.11	39.99	46.85	158.44
10	CH3	1	3	62.46	31.94	70.22	40.87	173.27
11	CH3	1	3	97.26	21.13	74.07	38.49	154.36
12	CH3	1	3	101.92	34.14	122.25	43.89	181.03
1	CH6	1	6	118.42	21.94	103.86	35.89	168.47
2	CH6	1	6	53.69	25.15	53.95	30.80	170.73
3	CH6	1	6	53.81	16.03	37.52	34.28	145.09
4	CH6	1	6	76.88	14.41	45.67	43.97	147.38
5	CH6	1	6	55.67	19.73	47.19	33.36	154.32
6	CH6	1	6	65.16	20.73	59.61	39.94	156.00
7	CH6	1	6	82.80	17.80	65.18	48.09	150.42
8	CH6	1	6	73.81	10.07	33.72	43.08	131.67
9	CH6	1	6	39.54	19.05	32.61	32.99	151.03

10	CH6	1	6	40.19	24.21	40.47	34.98	165.32
11	CH6	1	6	71.61	14.35	44.26	40.40	143.60
12	CH6	1	6	40.65	25.38	45.11	35.99	165.65
1	CH9	1	9	61.51	15.68	52.80	41.91	139.93
2	CH9	1	9	62.51	19.23	66.52	45.51	155.85
3	CH9	1	9	31.97	15.29	28.92	36.44	134.77
4	CH9	1	9	51.61	11.06	29.32	28.76	127.34
5	CH9	1	9	34.30	16.40	30.61	34.22	147.33
6	CH9	1	9	37.24	19.66	40.68	29.15	151.55
7	CH9	1	9	63.98	17.45	59.06	47.39	156.04
8	CH9	1	9	37.62	8.89	15.53	31.92	119.06
9	CH9	1	9	64.59	15.99	50.82	36.72	158.50
10	CH9	1	9	32.51	19.80	32.76	35.03	160.35
11	CH9	1	9	38.93	14.05	30.40	37.27	129.73
12	CH9	1	9	47.48	24.51	68.05	47.55	177.95
1	CH12	1	12	68.89	21.62	48.06	37.18	153.58
2	CH12	1	12	73.88	19.37	44.44	35.39	148.80
3	CH12	1	12	48.89	29.42	50.15	30.59	165.32
4	CH12	1	12	59.22	24.39	47.76	31.52	158.05
5	CH12	1	12	48.56	26.79	41.13	42.32	167.82
6	CH12	1	12	54.97	23.08	40.68	35.36	166.92
7	CH12	1	12	71.91	10.31	21.04	35.43	134.91
8	CH12	1	12	50.66	8.23	12.24	36.31	118.44
9	CH12	1	12	82.55	17.28	40.24	37.53	161.39
10	CH12	1	12	43.33	12.09	14.59	38.71	140.84
11	CH12	1	12	51.71	13.90	23.32	44.00	131.12
12	CH12	1	12	64.30	11.23	21.76	42.55	132.38

NO	GROUP	Challenge	Propolis	Cortisol	HLratio	MPO	MDA	
1	CNT	0	0	27.51	0.40	43.88	2.96	
2	CNT	0	0	33.39	0.41	38.59	3.07	
3	CNT	0	0	25.16	0.41	40.34	3.13	
4	CNT	0	0	21.01	0.38	38.84	2.91	
5	CNT	0	0	26.94	0.42	42.07	2.20	
6	CNT	0	0	27.96	0.43	42.36	3.03	
7	CNT	0	0	25.16	0.40	39.98	2.19	
8	CNT	0	0	22.01	0.44	42.30	2.80	
9	CNT	0	0	26.94	0.42	42.60	2.61	
10	CNT	0	0	27.96	0.40	43.74	2.55	
11	CNT	0	0	22.21	0.42	47.89	2.92	
12	CNT	0	0	31.47	0.43	41.14	3.05	
1	CH0	1	0	56.59	0.61	87.31	6.60	
2	CH0	1	0	62.37	0.62	85.95	4.60	
3	CH0	1	0	47.31	0.61	89.47	6.26	
4	CH0	1	0	56.81	0.62	85.55	7.26	
5	CH0	1	0	47.47	0.60	89.51	5.51	
6	CH0	1	0	50.02	0.61	83.77	4.55	
7	CH0	1	0	57.70	0.62	90.81	6.53	
8	CH0	1	0	63.01	0.62	85.95	5.59	
9	CH0	1	0	59.67	0.61	87.31	6.53	
10	CH0	1	0	55.11	0.61	84.23	6.37	
11	CH0	1	0	37.29	0.62	83.79	4.37	
12	CH0	1	0	58.80	0.62	85.53	6.09	
1	CH3	1	3	49.92	0.59	82.47	6.07	
2	CH3	1	3	55.71	0.58	80.25	4.73	
3	CH3	1	3	40.65	0.59	82.01	6.91	
4	CH3	1	3	36.81	0.59	82.43	5.18	
5	CH3	1	3	47.47	0.59	82.01	5.56	
6	CH3	1	3	50.02	0.60	84.19	3.65	
7	CH3	1	3	44.36	0.60	83.79	3.48	
8	CH3	1	3	29.68	0.58	80.71	5.12	
9	CH3	1	3	46.34	0.59	82.03	3.08	
10	CH3	1	3	55.11	0.60	84.23	4.45	
11	CH3	1	3	37.29	0.60	83.77	6.97	
12	CH3	1	3	58.80	0.60	84.19	7.26	
1	CH6	1	6	40.40	0.57	78.95	6.75	
2	CH6	1	6	46.23	0.57	78.49	3.08	
3	CH6	1	6	31.63	0.54	73.63	3.29	
4	CH6	1	6	27.33	0.57	78.51	4.41	
5	CH6	1	6	38.46	0.54	73.63	3.40	
6	CH6	1	6	41.21	0.53	71.45	4.10	
7	CH6	1	6	35.51	0.53	71.87	5.18	
8	CH6	1	6	20.70	0.54	73.23	4.54	
9	CH6	1	6	37.36	0.54	73.23	2.43	

10	CH6	1	6	45.92	0.55	75.43	2.40	
11	CH6	1	6	28.15	0.55	74.97	4.30	
12	CH6	1	6	49.99	0.53	71.47	2.56	
1	CH9	1	9	22.84	0.56	59.95	4.10	
2	CH9	1	9	30.70	0.54	62.15	4.02	
3	CH9	1	9	25.06	0.51	56.39	2.27	
4	CH9	1	9	16.14	0.52	59.45	3.47	
5	CH9	1	9	27.25	0.51	62.11	2.21	
6	CH9	1	9	30.04	0.56	61.71	2.41	
7	CH9	1	9	28.64	0.52	64.39	3.97	
8	CH9	1	9	11.43	0.54	59.93	2.51	
9	CH9	1	9	26.42	0.51	68.35	3.78	
10	CH9	1	9	30.28	0.56	67.45	1.93	
11	CH9	1	9	20.15	0.56	57.67	2.70	
12	CH9	1	9	45.95	0.48	61.71	3.08	
1	CH12	1	12	34.85	0.58	61.95	5.00	
2	CH12	1	12	29.73	0.60	63.65	5.22	
3	CH12	1	12	47.47	0.59	58.39	3.77	
4	CH12	1	12	39.46	0.58	60.95	4.37	
5	CH12	1	12	43.78	0.58	64.11	3.41	
6	CH12	1	12	43.66	0.50	63.21	3.91	
7	CH12	1	12	18.18	0.50	66.39	4.87	
8	CH12	1	12	13.30	0.52	61.43	3.71	
9	CH12	1	12	31.45	0.51	70.35	5.28	
10	CH12	1	12	21.61	0.50	68.95	2.83	
11	CH12	1	12	23.30	0.54	59.67	3.90	
12	CH12	1	12	19.97	0.50	63.21	4.58	

NO	GROUP	Challenge	Propolis	TAOC	TSOD	GSH	CAT	
1	CNT	0	0	1.68	6.19	16.08	50.96	
2	CNT	0	0	1.62	6.38	15.17	52.83	
3	CNT	0	0	1.17	6.58	16.21	47.93	
4	CNT	0	0	1.01	6.77	16.88	50.53	
5	CNT	0	0	1.33	6.96	17.24	52.80	
6	CNT	0	0	1.64	7.15	17.29	52.45	
7	CNT	0	0	2.23	7.34	15.83	54.73	
8	CNT	0	0	1.33	7.54	16.94	50.94	
9	CNT	0	0	1.12	7.73	16.80	58.10	
10	CNT	0	0	1.15	7.92	17.60	57.33	
11	CNT	0	0	1.65	9.13	15.84	49.02	
12	CNT	0	0	1.22	6.77	17.41	52.45	
1	CH0	1	0	1.19	2.83	12.07	35.28	
2	CH0	1	0	1.13	3.02	13.60	42.84	
3	CH0	1	0	1.19	3.22	12.14	37.96	
4	CH0	1	0	1.19	3.41	13.19	35.28	
5	CH0	1	0	1.13	3.60	11.79	43.52	
6	CH0	1	0	1.08	3.79	12.33	37.80	
7	CH0	1	0	1.30	3.98	10.94	35.31	
8	CH0	1	0	1.13	4.18	11.47	42.84	
9	CH0	1	0	1.19	4.37	12.13	37.80	
10	CH0	1	0	1.24	4.56	11.39	35.22	
11	CH0	1	0	1.30	5.77	12.92	42.84	
12	CH0	1	0	1.19	3.41	12.33	34.72	
1	CH3	1	3	1.19	4.37	14.90	40.41	
2	CH3	1	3	1.08	4.56	14.84	39.00	
3	CH3	1	3	1.13	5.77	14.12	42.28	
4	CH3	1	3	1.19	5.98	14.84	38.68	
5	CH3	1	3	1.13	6.18	14.12	35.75	
6	CH3	1	3	1.36	6.38	13.79	46.35	
7	CH3	1	3	1.19	6.59	13.85	41.06	
8	CH3	1	3	1.19	6.79	14.06	39.27	
9	CH3	1	3	1.13	7.00	14.06	38.35	
10	CH3	1	3	1.52	7.20	14.38	40.70	
11	CH3	1	3	1.30	7.40	14.31	39.04	
12	CH3	1	3	1.47	7.61	13.79	34.68	
1	CH6	1	6	1.24	7.40	15.43	38.10	
2	CH6	1	6	1.36	7.61	15.10	39.68	
3	CH6	1	6	1.30	6.30	15.36	38.18	
4	CH6	1	6	1.30	6.52	15.42	40.48	
5	CH6	1	6	1.24	6.73	15.36	42.36	
6	CH6	1	6	1.36	6.95	15.68	42.18	
7	CH6	1	6	1.36	7.16	15.62	41.86	
8	CH6	1	6	1.47	7.38	15.16	42.02	
9	CH6	1	6	1.30	7.60	15.36	40.41	

10	CH6	1	6	1.41	7.81	15.69	43.12	
11	CH6	1	6	1.36	8.03	15.62	46.18	
12	CH6	1	6	1.30	8.24	15.68	40.48	
1	CH9	1	9	1.47	8.78	16.14	38.81	
2	CH9	1	9	1.47	7.79	15.94	47.12	
3	CH9	1	9	1.30	7.03	16.46	41.75	
4	CH9	1	9	1.36	4.77	15.88	38.81	
5	CH9	1	9	1.36	7.39	16.47	47.87	
6	CH9	1	9	1.24	5.35	15.62	41.58	
7	CH9	1	9	1.36	9.02	16.66	38.84	
8	CH9	1	9	1.24	5.78	15.94	47.12	
9	CH9	1	9	1.41	8.25	16.14	41.58	
10	CH9	1	9	1.47	5.97	15.69	38.74	
11	CH9	1	9	1.47	7.36	15.62	47.12	
12	CH9	1	9	1.42	6.41	15.88	38.19	
1	CH12	1	12	1.35	8.07	13.72	43.88	
2	CH12	1	12	1.25	6.62	15.14	38.59	
3	CH12	1	12	1.20	6.47	13.99	40.34	
4	CH12	1	12	1.15	4.06	15.09	38.84	
5	CH12	1	12	1.25	6.80	14.00	42.07	
6	CH12	1	12	1.06	4.55	14.84	42.36	
7	CH12	1	12	1.25	8.30	15.33	39.98	
8	CH12	1	12	1.06	4.92	13.55	42.30	
9	CH12	1	12	1.30	7.59	14.85	42.60	
10	CH12	1	12	1.25	5.07	13.33	43.74	
11	CH12	1	12	1.35	6.77	14.37	47.89	
12	CH12	1	12	1.21	5.45	13.50	41.14	

NO	GROUP	Challenge	Propolis	PHG	LYS	ACH50	TIg	PBLP
1	CNT	0	0	16.77	33.6328	51.41	3.84	2.89
2	CNT	0	0	16.56	27.00425	51.76	3.92	2.97
3	CNT	0	0	15.55	31.78689	52.36	3.90	2.97
4	CNT	0	0	15.22	28.15001	53.73	3.91	3.00
5	CNT	0	0	16.45	30.67728	52.88	3.99	3.12
6	CNT	0	0	16.89	30.64113	53.23	4.00	3.11
7	CNT	0	0	15.51	33.18144	51.92	3.95	2.97
8	CNT	0	0	16.55	28.58689	50.13	3.99	3.02
9	CNT	0	0	16.66	33.71386	51.31	3.96	2.99
10	CNT	0	0	17.32	32.8516	49.78	3.93	3.00
11	CNT	0	0	16	29.89608	51.37	3.97	2.98
12	CNT	0	0	15.84	32.29033	53.64	3.95	3.00
1	CH0	1	0	9.154167	24.2164	21.94	1.67	1.1906
2	CH0	1	0	9.483333	23.70213	21.96	1.77	1.2278
3	CH0	1	0	9.479167	24.89345	21.76	1.71	1.2147
4	CH0	1	0	9.758333	24.27501	22.26	1.78	1.1788
5	CH0	1	0	9.020833	23.93864	21.54	1.71	1.1648
6	CH0	1	0	9.204167	23.12056	22.00	1.75	1.2006
7	CH0	1	0	9.045833	23.99072	22.32	1.79	1.2124
8	CH0	1	0	9.479167	25.69345	22.25	1.72	1.175
9	CH0	1	0	9.941667	25.85693	21.81	1.77	1.1989
10	CH0	1	0	8.966667	22.6258	21.49	1.68	1.1387
11	CH0	1	0	8.833333	23.54804	22.10	1.80	1.2154
12	CH0	1	0	9.6	25.94517	21.67	1.75	1.2502
1	CH3	1	3	9.85	24.30617	21.43	1.63	1.253356
2	CH3	1	3	9.854545	24.31768	20.97	1.67	1.340502
3	CH3	1	3	10.09091	24.9164	21.05	1.66	1.272462
4	CH3	1	3	9.6	23.67292	21.79	1.67	1.211302
5	CH3	1	3	9.640909	23.77654	21.35	1.71	1.407524
6	CH3	1	3	9.75	24.05287	20.94	1.71	1.208668
7	CH3	1	3	9.527273	26.4887	20.99	1.69	1.222994
8	CH3	1	3	9.690909	23.90319	21.37	1.70	1.35812
9	CH3	1	3	9.631818	32.75352	21.53	1.69	1.248174
10	CH3	1	3	9.677273	23.86865	21.96	1.68	1.160688
11	CH3	1	3	9.531818	23.50021	21.36	1.70	1.42191
12	CH3	1	3	9.581818	23.62687	21.87	1.69	1.264836
1	CH6	1	6	10.24	27.72269	33.57	2.30	1.5156
2	CH6	1	6	10.355	28.01182	30.48	2.41	1.5054
3	CH6	1	6	10.21	27.64727	30.83	2.32	1.5222
4	CH6	1	6	10.16	27.52156	31.64	2.37	1.4796
5	CH6	1	6	10.225	27.68498	32.78	2.43	1.5367
6	CH6	1	6	10.43	28.20038	31.34	2.42	1.5069
7	CH6	1	6	10.33	27.94896	30.57	2.39	1.4221
8	CH6	1	6	10.11	27.39585	32.82	2.27	1.4594
9	CH6	1	6	10.39	28.09981	31.86	2.26	1.4505

10	CH6	1	6	10.27	27.79811	32.61	2.33	1.4566
11	CH6	1	6	10.21	27.64727	33.54	2.37	1.4943
12	CH6	1	6	10.47	28.30094	33.23	2.36	1.5001
1	CH9	1	9	14.06667	31.32391	36.14	3.32	1.824668
2	CH9	1	9	13.61333	30.3854	36.18	3.39	1.855756
3	CH9	1	9	14.05333	31.29631	35.84	3.35	1.879811
4	CH9	1	9	13.74667	30.66143	36.67	3.38	1.805099
5	CH9	1	9	13.50667	30.16457	35.48	3.29	1.8297
6	CH9	1	9	13.61333	30.3854	36.23	3.33	1.885796
7	CH9	1	9	13.24667	29.6263	36.75	3.07	1.856159
8	CH9	1	9	13.46	30.06795	36.64	3.09	1.854503
9	CH9	1	9	13.46667	30.08176	35.93	3.09	1.793727
10	CH9	1	9	13.76	30.68903	35.39	3.23	1.779661
11	CH9	1	9	13.62667	30.413	36.39	3.17	1.869621
12	CH9	1	9	13.50667	30.16457	35.69	3.14	1.89442
1	CH12	1	12	9.914286	29.55167	24.40	2.65	1.6585
2	CH12	1	12	10.30476	30.18109	23.83	2.71	1.6965
3	CH12	1	12	9.914286	29.55167	24.55	2.68	1.6764
4	CH12	1	12	9.957143	29.62076	23.54	2.70	1.6889
5	CH12	1	12	10.09524	29.84335	23.28	2.63	1.6446
6	CH12	1	12	10.12857	29.89708	23.21	2.66	1.6646
7	CH12	1	12	9.719048	27.81735	23.13	2.45	1.5333
8	CH12	1	12	9.633333	27.70421	23.35	2.48	1.5472
9	CH12	1	12	9.857143	27.99962	21.86	2.47	1.544
10	CH12	1	12	9.819048	27.94934	22.95	2.59	1.6167
11	CH12	1	12	9.77619	27.89277	22.33	2.53	1.5842
12	CH12	1	12	9.914286	28.07505	23.60	2.51	1.5679
